# Coil Embolization of a Portosystemic Shunt Presenting as a Varicocele

**DOI:** 10.14309/crj.0000000000001218

**Published:** 2024-09-20

**Authors:** Vincent Galate, Ian Kozlowski, Carmen Vogt, Ryan Ash, Adam Alli, Aaron Rohr

**Affiliations:** 1 Department of Radiology, University of Kansas Medical Center, Kansas City, KS; 2 University of Nebraska Medical Center College of Medicine, Omaha, NE

## CASE REPORT

**Figure 1.**
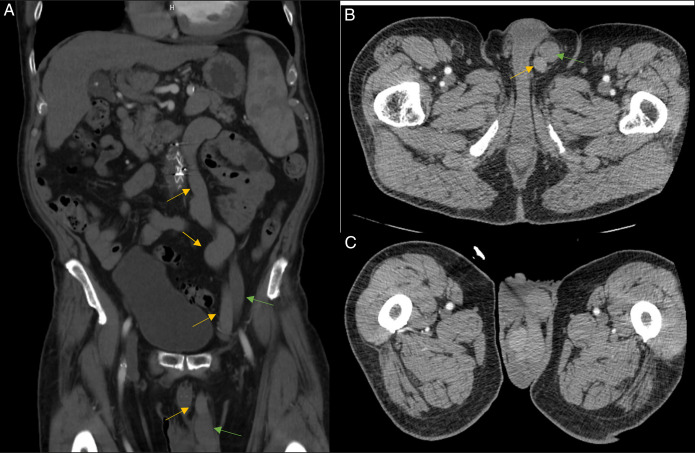
(A) Coronal view of abdominal/pelvic CT scan demonstrating dilated portal venous system vein (yellow arrows) and dilated central system vein (green arrows). (B) Axial view of abdominal/pelvic CT scan demonstrating portosystemic shunt. (C) Axial view of abdominal/pelvic CT scan showing dilated portal venous system (yellow arrow) and dilated vena cava system (green arrow). CT, computed tomography.

**Figure 2.**
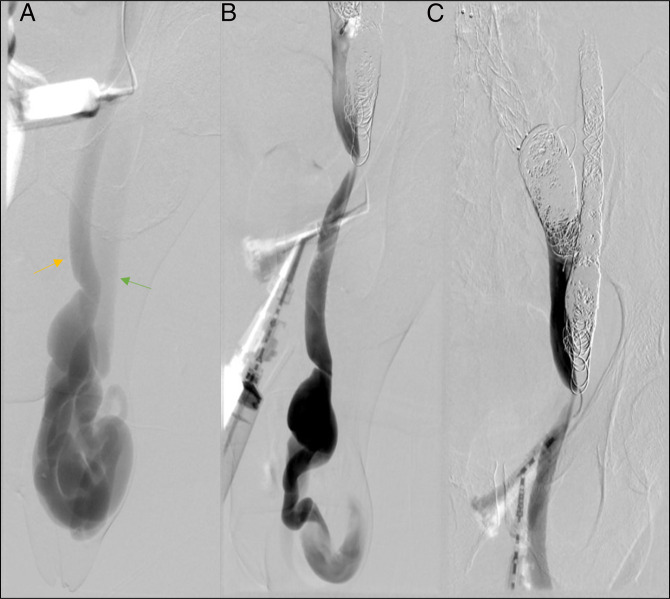
(A) Venography demonstrating portosystemic shunt (yellow arrow portal system, green arrow central venous systems). (B) Venography of portosystemic shunt postcoiling with decreased PSS filling. (C) Postcoiled venography of portosystemic shunt showing stasis of flow. PSS, portosystemic shunt.

In patients with portal hypertension, a portosystemic shunt may develop, redirecting blood flow from the portal system to the systemic venous system. These patients are at an increased risk of hepatic encephalopathy due to increased blood flow circumventing hepatic filtration through the portal system. We present a case of a 76-year-old man with alcoholic cirrhosis and portal hypertension who subsequently developed a portosystemic shunt presenting as a varicocele (Figure 1). He failed conservative management including scrotal support and decompression. Transjugular intrahepatic portosystemic shunt creation was discussed, but he lacked additional symptoms and wanted to avoid placing a shunt. He elected to undergo endovascular coil embolization. Access was gained through the internal jugular vein into the left gonadal vein and through the portal varix by direct micropuncture near the left inguinal ligament. The patient was treated with coil embolization through each access site. Post-treatment venography through the varix demonstrated complete stasis of flow (Figure 2). A 1-week follow-up discussion with the patient described near-complete resolution of pain associated with the shunt and decreased volume occupying the scrotum. We suggest that a patient with a varicocele in setting of liver cirrhosis undergo further workup to aid in treatment planning.

## DISCLOSURES

Author contributions: V. Galate wrote the manuscript. I. Kozlowski, C. Vogt, R. Ash, and A. Alli revised the final manuscript. A. Rohr revised the final manuscript and is the article guarantor.

Financial disclosure: None to report.

Informed consent was obtained for this case report.

